# Feasibility of an implementation strategy for preventing falls in homecare services

**DOI:** 10.1186/s43058-024-00615-7

**Published:** 2024-07-19

**Authors:** Siv Linnerud, Linda Aimée Hartford Kvæl, Maria Bjerk, Kristin Taraldsen, Dawn A. Skelton, Therese Brovold

**Affiliations:** 1 Department of Rehabilitation Science and Health Technology, Faculty of Health Sciences, OsloMet - Oslo Metropolitan University, St. Olavs plass, P.O. Box 4, N-0130 Oslo, Norway; 2 Department of Housing and Ageing Research, Norwegian Social Science, OsloMet - Oslo Metropolitan University, Olavs plass, P.O. Box 4 St, N-0130 Oslo, Norway; 3 Division for Health Services, Norwegian Institute of Public Health, Oslo, Norway; 4 Research Centre for Health (ReaCH), Department of Physiotherapy and Paramedicine, School of Health & Life Sciences, Glasgow Caledonian University, Glasgow, UK

**Keywords:** Implementation science, Falls prevention, Older adults, National recommendations, Process evaluation

## Abstract

**Background:**

Falls among older adults represent a major health hazard across the world. In 2022, the World Falls Guidelines was published, summarising research evidence and expert recommendations on how to prevent falls, but we need more knowledge on how the evidence can be successfully implemented into routine practice. In this study we used an implementation strategy co-created by healthcare providers, older adults who had fallen and researchers, to facilitate uptake of fall prevention recommendations. This current study aimed to evaluate the feasibility of this co-created implementation strategy in homecare services and provide information on the intervention and measurements for a full-scale cluster-randomized trial.

**Methods:**

This study was a single-armed feasibility study with an embedded mixed-method approach completed in two city districts of Oslo, Norway, over a period of ten weeks. The co-created implementation strategy consists of a package for implementing national recommendations for preventing falls, empowering leaders to facilitate implementation, establish implementation teams, competence improvement and implementation support. City districts established implementation teams who were responsible for the implementation. Feasibility was assessed both qualitatively and quantitatively, using focus group interviews with implementation team members and individual interviews with leaders and staff members and the Feasibility of Intervention Measure (FIM). Qualitative data were analysed using thematic analysis and the Normalisation Process Theory.

**Results:**

Qualitative data were collected from 19 participants: 12 implementation team members, 2 leaders and 5 staff members. 8 of the implementation team members responded to FIM. The analysis revealed four themes: 1) Fostering consensus through tailored implementation and discussions on fall prevention, 2) The importance of multi-level and interdisciplinary collaboration in fall prevention implementation, 3) Minimizing perceived time usage through utilization of existing areas for implementation activities, and 4) Reflective monitoring demonstrates the importance of facilitation and structure in the implementation strategy. For FIM, there were a high level of agreement related to how implementable, possible, doable, and easy to use the implementation strategy was.

**Conclusions:**

Overall, we found the implementation strategy to be feasible to enhance uptake of fall prevention recommendations in the Norwegian homecare services. To succeed with the implementation, a dedicated implementation team should receive support through the implementation process, they should choose small implementation activities to enhance fall prevention competence and managers should possess implementation knowledge.

**Trial registration:**

The trial is registered in the Open Science Registry: https://doi.org/10.17605/OSF.IO/2JFHV Registered: January 11, 2023.

**Supplementary Information:**

The online version contains supplementary material available at 10.1186/s43058-024-00615-7.

Contribution to the literature

- Reporting on the feasibility of an implementation strategy to implement fall prevention recommendations provides information that can be useful for scaling up implementation of fall prevention recommendations and implementing in other settings.
- This study demonstrates a feasible way to implement fall prevention recommendations, establishing implementation teams, empowering leaders to facilitate implementation, enhancing healthcare providers competence, and providing implementation support.
- Using the Normalisation Process Theory to analyse the results provides an understanding of how the implementation strategy is accepted and integrated into homecare services.

## Background

Falls among older adults represent the second leading cause of unintentional injury deaths across the world [1]. One-third of older adults’ experience a fall every year [2]. Repeated falls among older adults is often a result of other health-related issues such as co-morbidity, impaired balance, muscle weakness or frailty [3]. Falls among older adults occur in various settings, including but not limited to, homes, hospitals and community spaces. The World Falls Guidelines summarise previous research evidence and provides expert recommendations on how to identify and assess risk factors and manage effective interventions to prevent falls [4]. The guidelines recommend yearly screening of older adults above 65 years to identify falls, multifactorial assessment of persons at risk, along with specific recommendations on interventions that should be offered due to level of risk [4]. In 2023, the Norwegian Directorate of Health developed national recommendations to prevent falls among older adults [5]. The national recommendations are building on the Worlds Falls Guidelines and state who holds the responsibility for preventing falls, training of health care providers, how to identify and measure falls, as well as recommended interventions based on context and the persons level of risk. The guideline provides recommendations that take into account the diverse context in which falls occur. In this study we used a draft of the recommendations as they were not published at the time of the study. None of the city districts had implemented the recommendation previously, however they had routines for risk assessment of service users who had experienced falls. The city districts also had balance-and strength exercises for groups and home safety visits in place.

Despite substantial trial evidence on effective ways to prevent falls, such as providing strength and balance training to older adults [2], improving home safety [6] or reviewing medication use [4], we still need implementation studies that can provide knowledge on how the evidence can be successfully implemented into routine practice. Implementation science recommends using specific implementation strategies, or methods to increase uptake in practice [7]. To strengthen the success of strategies, they should target barriers and facilitators [8, 9], and be tailored to local context [10]. Previously strategies used to implement fall prevention targeting community-dwelling older people have been strategies aiming to train and educate stakeholders [11–13], and providing tailoring and individualization [11]. However, as far as we know, few studies have evaluated the effect of implementation strategies used to enhance fall prevention recommendations in community care.

In a previous study, we used co-creation to develop an implementation strategy to facilitate uptake of fall prevention [14]. In this study healthcare providers in homecare services, older adults who had experienced falls and researchers identified barriers and facilitators for implementation of fall prevention recommendations in local homecare services. Examples of identified barriers were lack of acknowledgement and prioritizing of fall preventions, time and resources, lack of fall prevention knowledge, and systems for implementation. The barriers indicated a need for strategies targeting the empowerment of leaders to facilitate implementation, establishing implementation teams to facilitate the implementation process, tailoring dual competence improvement on both fall prevention and implementation, and the need for providing implementation support. These four components should be included in implementation strategies targeting implementation of fall prevention in the setting of homecare services [15]. The current study suggests how to implement this co-created implementation strategy and evaluates the feasibility through process evaluation.

### Theoretical framework

The Normalisation Process Theory (NPT) is an implementation theory identifying, describing and explaining mechanisms promoting the implementation of complex interventions [16]. The theory was chosen since it provides understanding of how interventions are accepted and integrated into the social context of practice, and is suitable for complex interventions with multiple stakeholders [17]. NPT consists of four components: coherence, cognitive participation, collective action, and reflexive monitoring. Coherence refers to a collective meaning and understanding of the intervention. Cognitive participation reflects the collective investment of commitment and engagement. Collective action represents the investment of effort and resources to make the intervention function, while reflexive monitoring represents the assessment of benefits [18]. In this study we used the theory as an analytic tool to interpret integration and acceptance of the implementation strategy into routine practice, to accomplish normalization.

### Aim of the study

This study aimed to evaluate the feasibility of a co-created implementation strategy in homecare services targeting implementation of fall prevention recommendations.

## Methods

### Design

This was a single-armed feasibility study evaluating the feasibility of an implementation strategy in homecare services [19]. The study aimed to provide relevant information for a full-scale cluster-randomized trial within the FALLPREVENT project (Implementation of evidence-based, fall-prevention programmes in the health care services: Quality, competency and effectiveness) [20], where the feasibility design provides assessment on how realistic it is to carry out the implementation strategy and determine its viability. We used both individual interviews and a questionnaire to assess feasibility, and to test if the questionnaire could be used in large scale study. Exploring this on a small scale before initiating a full-scale trial is essential to avoid wasting time or money for the services. The study adheres to the Standards for Reporting Implementation Studies (StaRI) statement [21].

### Context

This study was carried out in homecare services in two city districts of Oslo municipality, Norway. Oslo is the largest city and capitol of Norway, with 15 city districts including 700 000 inhabitants. The city districts are mainly self-governing and responsible for primary health care. Primary health care includes homecare services provided to the inhabitants in their homes, in addition to nursing homes and other living facilities [22]. The homecare services provide services such as home nursing, rehabilitation, and practical assistance, delivered by multidisciplinary healthcare providers: nurses, physiotherapists, occupational therapists, nursing assistants, and unskilled employees [23].

### Participants

The participants in this study included 19 healthcare providers, with different professions from the homecare services of two city districts of Oslo municipality. The city districts established implementation teams with hand-picked members (*n*=12), based on their personal qualities, their role in the organisation and their previous experience with fall prevention and implementation. A mix of professionals from different levels of the organisation made up the implementation teams, including healthcare providers working in clinical practice, mid-level managers, such as team managers, and high-level managers, such as department managers. Additionally, we included managers not participating in the implementation teams (*n*=2) and a selection of staff members (*n*=5) who had received implementation actions provided by the implementation team. Recruitment was carried out by the management in each of the city districts. City-districts did not receive any funding for participating in this study, but were invited to a small event with cake and treats at the end of the study.

### The implementation strategy

The co-created implementation strategy consists of a package for implementing recommendations for preventing falls, empowering leaders to facilitate implementation, establishing implementation teams, tailor dual competence improvement and provide implementation support (Table 1). The intervention period was 10 weeks and the strategy comprised three phases inspired by the phases in the process domain of the 2009 version of the Consolidating Framework of Implementation Research (CFIR) [24]. The first phase involved identifying a structure for implementation, including establishing the implementation teams. The second phase included preparation for the implementation, and the third phase was the actual implementation. Throughout the process, the implementation teams were responsible for the implementation. The city districts received support and facilitation through all phases by two of the authors (SL and MB).

**Table 1:** Components of the implementation strategy

**Component**	**Strategy**	**Actors**	**Actions**	**Targets of the action**	**Temporality**	**Dose**	**Implementation outcome affected**
Empower leaders to facilitate implementation	Leader commitment	Facilitators	Leader commitment was built through local meetings with managers during phase 1, and by establishing participation of managers in the implementation teams.	Leaders and managers in city districts	Phase 1	Local meeting with managers	Feasibility
	Train leaders in implementation	Facilitators	Leaders were trained in their role in implementation	Leaders and managers in city districts	Phase 1	One 3-h seminar with managers	Feasibility
Tailor dual competence improvement	Train implementation teams in implementation process	Facilitators	Implementation teams were trained in the steps of the Action cycle of Knowledge-to-Action. To support each step, the Norwegian-adapted version of the implementation toolkit Implementation of Best Practice Guidelines [19].	Implementation teams	Phase 2	Four 3-h workshops with each implementation teams	Feasibility
	Make training dynamic	Facilitators	The information delivery methods were varied with use of discussion, quiz and reflection tasks to help the training to be interactive.	Implementation teams	Phase 2 and 3	Four 3-h workshops with each implementation teams	Feasibility
	Develop and distribute educational material	Facilitators	Supporting materials for the implementation teams to use in local implementation	Implementation teams	Phase 2 and 3	PowerPoint presentations on fall prevention, local fall rates and statistics on fall related injuries and cost, fall prevention quiz and fall related patient case.	Feasibility
Provide implementation support	Facilitation and support	Facilitators	City districts and managers received support in establishing the implementation teams in the first phase. Facilitation was provided to the implementation team for the implementation process. The teams also received support through meetings with the facilitators during local implementation.	Implementation teams and managers	Phase 1, 2 and 3	Four 3-h workshops with the implementation teams, meetings with managers, and mail support when needed.	Feasibility

- Phase 1: Establishing a structure for implementation

In the first phase, each city district identified and established a team that would be responsible for the implementation. The facilitators offered guidance in the process, providing advice on important roles and qualifications relevant in the teams. Each team consisted of six hand-picked members, involving staff from different levels of the organization such as managers, team nurses, and physiotherapists. During this phase, one city district also requested enhancement of implementation knowledge among managers, which was provided to mangers from both city districts, through a 3-hour seminar. This seminar for managers included an introduction to implementation science, previous knowledge in the field and discussion on their experiences from practice.

- Phase 2: Preparing for implementation

The second phase was carried out within each city district. The implementation teams attended three workshops of three hours each, including homework between the workshops. The content of the workshop followed the steps of the action cycle of Knowledge to Action model [25], accompanied by the Norwegian version of the implementation toolkit “Implementation of best Practice Guidelines”. During this phase, the teams received implementation support and were offered guidance on the activities. To support the teams with improving fall prevention competence in the city districts, they received a toolbox with supporting materials, such as a fall prevention quiz and PowerPoint presentations etc. The content of the toolbox was developed by the facilitators, based on what the implementation teams requested.

- Phase 3: Implementation

The third phase involved the implementation of the national recommendations on fall prevention into routine practice, where the teams tested their chosen implementation strategies within their city district. During this phase, support was given based on the teams’ expressed needs. For example, one of the teams requested regular follow-up by emails every second week, while the other team wanted a digital follow-up meeting halfway through phase three.

### Data collection and outcome measures

Feasibility, the extent to which the implementation strategy can be carried out successfully in the homecare services [26], was assessed both qualitatively and quantitatively. Two focus group interviews with a total of 6 implementation team members and semi-structured individual interviews with managers (n=4) and staff (n=5) were conducted. The sample size was informed by information power as described by Malterud, regarding the specificity of participants, breadth of aim, quality of dialogue, application of theory, and strategy chosen for the analysis [27]. All interviews were performed shortly after the end of the implementation period (see Interview guide in additional file 1) and lasted approximately one hour. Focus group interviews were conducted at Oslo Metropolitan University, facilitated by two researchers (KT and TB) who had not participated in the study (see topic guide in additional file 2). Managerial interviews were conducted digitally using Zoom, while staff interviews took place at their respective workplace. Individual interviews were conducted by the main author (SL). In each workshop, participating observation (e.g., engagement and gestures) and observation notes were also made, to be able to make necessary adjustments to the content.

The three authors primary engaged in the analysis had clinical backgrounds in nursing (SL) and physiotherapy (LAHK and TB). They possessed extensive clinical experience, with two of them having worked in community care and homecare services (SL and LAHK). Our preconceptions illustrated challenges in engaging the implementation teams in the implementation process and lack of leadership from mangers.

To assess the feasibility of the implementation strategy quantitatively, the Norwegian version of the Feasibility of Intervention Measure (FIM) was used. FIM is a four-item questionnaire with a five-point response scale (from 1 Completely disagree to 5 Completely agree), specially developed to assess feasibility as an implementation outcome. The questionnaire has displayed acceptable psychometric properties [28]. The implementation team members responded anonymously to the electronic questionnaire after the second phase, preparing for implementation.

### Analysis

Individual interviews and focus group interviews were transcribed verbatim, checked, and corrected by the first author. The transcripts were then, uploaded into HyperResearch 4.5.3 for data management and analysed in accordance to Braun and Clarke`s description of thematic analysis [29]. First, three of the authors (SL, LAHK and TB) independently read all transcriptions to become familiar with the material. They proceeded to independently take notes on insights and patterns from the transcripts. Subsequently, they engaged in discussion to reach consensus on the content. The first author coded the transcripts by using code labels closely linked to the meaning of the quotes, organized codes with similar content into groups and generated initial themes. Groups and initial themes were then presented and discussed with two of the other authors (LAHK and TB). Then, in phase three, NPT was used as a theoretical lens to understand how the implementation of fall prevention practice in the current study is embedded and integrated into the social context of the two homecare services [30]. Codes and initial themes, organized within the constructs of NPT, were reviewed by three authors (SL, LAHK and TB) before being refined and delineated into four main themes. In cases where themes overlapped with more than one NPT construct, a choice of best fit was made. The content of the final themes was discussed with all authors. Observations made through the phases of implementation were used to support, explain, or differentiate the results.

Results from FIM were analysed using simple statistics within Microsoft Excel, providing descriptive statistics, such as distribution of responses and means, for each of the question.

## Results

The implementation strategy required city districts to tailor the implementation process based on local needs and context. Each city district established individual aims for the implementation. The implementation teams assessed for local barriers and facilitators, tailored implementation strategies to fit with their local context and created a plan for the implementation. Aims, barriers and implementation strategies chosen by the teams within the two city districts, are presented in Table 2.

**Table 2:** Aims, barriers and implementation strategies of each city district

	**City district #1**	**City district #2**
**Aim for implementation**	- All service users receive yearly screening of falls through questions on falls once a year - Service users who have fallen receive a multifactorial assessment - Users with a high risk of falling, receive personalized interventions discussed in multidisciplinary meetings - Fall prevention education and information provided to staff	- Staff have knowledge about the local fall prevention pathway - Falls registered correctly
**Local barriers**	- Knowledge and beliefs about the intervention - Access to knowledge and information - Individual stage of Change - Compatibility - Goals and Feedback	- Knowledge and beliefs about the intervention - Access to knowledge and information - Individual stage of Change - Compatibility - Leadership engagement
**Local tailored strategies**	Train and educate stakeholders: - Provide fall prevention education during lunches - Using webinars about fall prevention - Adjust the education shedule for newly employed staff to include fall prevention Remind clinicians: - Register questions to identify falls as standard yearly activity in all service users journals - Register questions to identify falls as a standard task for all new service users	Train and educate stakeholders: - Education on fall prevention during morning meetings - One-on-one follow-up on the fall prevention pathway and correct registration on falls - Adjust the education schedule for newly employed staff to include fall prevention

### Qualitative results

The analysis revealed the following four themes: 1) Fostering consensus through tailored implementation and discussions on fall prevention, 2) The importance of multi-level and interdisciplinary collaboration in fall prevention implementation, 3) Minimizing perceived time usage through utilization of existing areas for implementation activities, and 4) Reflective monitoring demonstrates the importance of facilitation and structure in the implementation strategy.

### Fostering consensus through tailored implementation and discussions on fall prevention

Emphasising a common understanding reflects both the implementation teams shared understanding of the implementation strategy and the consensus on the importance of fall prevention achieved among staff members in the city districts.

Overall, implementation team members, leaders and staff members underscored how important it was to achieve a common understanding, both related to what is to be implemented, why this is important and how it should be done. However, during the first workshop, we observed an insecurity among participants in the implementation teams regarding the purpose of the workshop and their role in the implementation. This was also confirmed when interviewing the team members, who described a sense of unpreparedness and uncertainty, despite being positive towards the engagement. One of the team members explained:

*To be honest, I didn’t know what I was participating in. I had no idea, I thought I was going to a lecture when I arrived for the first workshop. However, the first workshop clarified a lot ...* (Participant 09)

Despite the lack of information beforehand, we observed during the first workshop that the implementation teams achieved a shared understanding of the implementation strategy, and the importance of preventing falls among community-dwelling older adults.

However, through the implementation period we noted that some of the team members did not attend at all the workshops, even though they had originally shown interest in participating. One team member attributed this to sickness absenteeism, while others cited competing priorities as the reason for their absence.

Furthermore, the implementation strategy required a way of working that differed from how they usually worked. Initially, participants in the implementation teams found it novel and challenging to tailor the implementation to the local context themselves. One of the team members stated:

*It’s up to us to identify barriers and facilitators and figure out how to implement… and it was a little, not demotivating, but it would have been easier having a recipe to follow… and knowing what works and what doesn’t work* (Participant 07)

Even though the implementation strategy represented a new way of working for the teams, they quickly recognised the importance of tailoring it to the local context. Participants described tailoring as a mean of achieving ownership and building capability among the members of the implementation team.

During the implementation phase, the teams quickly experienced a shared understanding of preventing falls among other staff members. Staff members stated the importance of grasping the rationale behind preventing falls as an important aim. Achieving this shared understanding influenced the adherence of fall prevention recommendations among the staff and motivated them to prioritise fall prevention activities in a hectic workday. One of the staff members said:

*The workdays here are so hectic. So, for me it is important to understand why I am doing something. If not, I might not do it because I don’t have time for it. But if I understand the importance, I might find time to do it after all* (Participant 14).

Another staff member shared that she had always been particularly observant about falls among the service users. However, she noted a noticeable shift among her co-workers during the implementation period, which underscores that the implementation strategy had placed falls on the agenda. She said:

*Now everybody talks a lot about falls […] The whole department, the coordinating nurse talks about falls in our daily morning meeting. Remember to write it as an OU message [electronic message to other healthcare providers] if you don’t have time to come in [to the office] and do the registration on a computer. So, it’s easier for us, just sending an OU message right away, than to find a computer.* (Participant 15)

### The importance of multi-level and interdisciplinary collaboration in fall prevention implementation

Members of the implementation teams were hand-picked based on their education, experience, and role in the city district, and had the responsibility for the implementation. Throughout the study, the city districts invested effort and resources through the implementation teams, who were the key people driving the process forward. The commitment of the implementation teams promoted the implementation process and led to further engagement in the city districts. The participants expressed the importance of having a team consisting of members carefully chosen based on their role in the organisation, skills, and competence. One of the team members said:

*We are probably hand-picked because we are easy to collaborate with […] We don’t see it as extra work, but... an opportunity to make a change* (Participant 06).

Several of the implementation team members thought it was positive being part of a group consisting of different professions, both because it represented the diversity in healthcare providers working with fall prevention in practice, but also because they could contribute with different perspectives.

Having an implementation team comprising members with different professions and competencies also helped illustrate the importance of fall prevention being a multidisciplinary task. Different members made unique different contributions in the implementation process, and we observed the teams strategically using the diverse competencies of the team members. As an example, the physiotherapists used their expertise in falls and fall prevention to educate other staff members, while the team nurse incorporated fall prevention tasks into the worklists of the staff she supervised. One of the members said:

*We made a plan on what to do and when, and then we shared the responsibility. Whom did what, so everyone knew what to do….It was easy to agree on and makes it easier to take responsibility* (Participant 08)

The implementation teams in both city districts included a manager, and we observed that these two managers assumed different roles in the teams. In one of the teams, the manager took on an active leadership role and led the process, while in the other team, the manager adopted a more neutral role, thereby delegating more responsibility to the other team members. The team members in both teams expressed satisfaction with how the manager filled the role in their team. All participants in the implementation teams highlighted the importance of including the manager in the team. Including mangers in the teams verified leader commitment, as the participants in the teams found the managers provided necessary support during the implementation process. One of the team members explained:

*If we did face any challenges, it was easy to reach out to the manager in the implementation team. Then the manager could talk to those involved and figure out how to solve it.* (Participant 08).

The managers agreed that they were satisfied with their own involvement as it helped them to stay informed. In one of the city districts, involvement of managers was highlighted as an important strategy to gain the desired progress in the process.

### Minimizing perceived time usage through utilization of existing areas for implementation activities

Overall, both city districts used implementation strategies targeting training and education of staff and increasing the focus on fall prevention through low use of time and resources. To implement fall prevention recommendations in the city districts, the participants strongly agreed that fall prevention needed to be put on the daily agenda. Both implementation teams used already existing meeting arenas and they described this as a success because the team and the rest of the staff could all save time by sharing information more efficiently. One city district used an existing “focus of the week/month white board” to highlight fall prevention as a collective aim for the implementation period. The other city district used the daily morning meeting for the same purpose, actively reminding staff about activities targeting fall prevention. One of the staff said:

*Identification of falls has been the focus of the month. It has been written on our focus board, that we are focusing on identifying falls. It makes it easy to ask someone “what is this” […] I think it’s a good way to create focus, then you can’t say you haven’t heard about it. When it is right there on the wall, very visual, when you enter the room (*Participant 14).

Participants also described using existing arenas for training of the staff and found this not to be too time consuming if integrated into everyday work. These meeting points were also a platform for sharing information regarding the prevalence, cost, and risk factors associated with fall prevention.

Furthermore, most of the involved managers, staff and team members said they preferred the relatively short length of the implementation period. The staff described hectic days in the homecare service, with a variety of ongoing tasks as well as new activities that they needed to handle during a workday. Keeping focus over time was challenging and they experienced loss of focus after some time. The short implementation period made them keep high attention on the topic for a period but would require repetition regularly when the attention dropped. One of the staff expressed:

*I don’t know how long it takes to get people to do things differently, but I think it’s important with repetition. Not every day, but repeating things with a certain duration.* (Participant 14).

The time invested in the implementation primarily involved the participation of implementation team members. They attended workshops to prepare for the implementation and led implementation activities in each city district. This investment of time from team members was deemed essential for integration into existing work processes.

The two city districts chose different implementation activities for their city district, and the duration of extra time spent on implementation therefore differed. Regardless of this, both teams agreed that the implementation intervention was manageable and did not require much time and resources. One of the participants in the implementation teams said:

*The only thing we spent extra time doing, was the nine hours we spent on the three workshops with the facilitators. It took some time; it was three half days. But that’s what you need for the planning, so it was time well spent... That’s the time you need to make room for and prioritise if you want to participate in it.* (Participant 06).

Despite having this shared experience on little use of time, in one of the city districts they chose to use the patient journal as a task manager for asking all service users about falls. This required a manual update of all the journals and was considered a time-consuming process not part of the predicted timeline, even though it was a one-time task. One of the team members said:

*So, it was an intense... a bit more time-consuming than I had imagined. So, when we are going to implement this at the other departments, I would recommend releasing someone from their regular tasks to do it.* (Participant 09)

Overall, the districts planned implementation activities that would not impede time spent on direct patient work, and the staff that received the fall prevention training or activities did not feel they were time consuming or resource demanding. This underscores the importance of utilizing existing platforms to seamlessly integrate the implementation into existing workflows, rather the treating it as additional work

### Reflective monitoring demonstrates the importance of facilitation and structure in the implementation strategy

Overall, all participants expressed satisfaction with the implementation intervention. When the members of the implementation teams reflected on what made the intervention a success, the majority highlighted the structure of the implementation strategy. Dedicated time through the workshops helped the implementation team members to maintain progress of the implementation activities and receiving support was important for the teams to get things done. One of the team members expressed:

*It was very useful with the support, and having regular workshops demanded progress. Even being on a larger scale, it’s a question about capacity within it* (Participant 06)

One of the managers expressed the importance of having increased the implementation knowledge among other managers. The implementation knowledge was valuable because it could be applied to other implementation tasks beyond fall prevention recommendations. The seminar held for managers was highlighted as important for creating a common understanding for implementation, providing a platform for discussing both successful and unsuccessful experiences with implementation. One of the managers stated:

*What is most useful, is to emphasize how to succeed with implementation among managers. That’s what was most useful for me as a manager, achieving a common understanding [for implementation]* (Participant 01)

One of the staff who had started to ask service users about falls, highlighted how this led to more self-reflection. She was surprised by the answers she received from the service users; they did not respond how she expected them to respond. Asking this question worked for her as an invitation to more information about the users and led to a deeper conversation. She said:

*I became more aware of the differences among service users, and how different they think about fall risks and what fall prevention actually is* (Participant 14)

The participants also described an increased understanding about fall prevention, where staff expressed gaining a better understanding of risk factors and consequences of falls. In one of the city districts they stated that they had seen an increase in referrals to physiotherapists for fall risk assessment and environmental modification among older adults with fear of falling, such as removal of rugs. One of the staff said:

*They gave us information about things we could do to prevent falls and how to evaluate risk of falling. […] Just during the last week, I have informed the physiotherapist about three or four service users with high risk of falling.* (Participant 13)

The facilitators also made some smaller changes to the content of the workshops during the study, based on observations. The changes were mainly related to duration of time used on the different tasks, or changes in how the tasks were presented.

### Feasibility implementation measure

Eight of the implementation team members responded to the FIM, leaving a response rate for 66%. The participants level of agreement related to how implementable, possible, doable, and easy to use the implementation strategy was, are presented in Table 3. The results indicate a high level of agreement on all questions, finding the implementation strategy to be both implementable, possible and doable. While for the last question one participant neither agreed nor disagreed on how easy to use the implementation strategy seemed.

**Table 3:** Results of implementation team members response to feasibility implementation measure

	Completely disagree % (n)	Disagree % (n)	Neither agree nor disagree % (n)	Agree % (n)	Completely agree % (n)
1. The implementation strategy seems implementable.	0 (0)	0 (0)	0 (0)	87.5 (7)	12.5 (1)
2. The implementation strategy seems possible.	0 (0)	0 (0)	0 (0)	75 (6)	25 (2)
3. The implementation strategy seems doable.	0 (0)	0 (0)	0 (0)	100 (8)	0 (0)
4. The implementation strategy seems easy to use.	0 (0)	0 (0)	12.5 (1)	87.5 (7)	0 (0)

## Discussion

This study assessed feasibility of an implementation strategy aiming to embed Norwegian fall prevention recommendations into homecare services, providing information for a full-scale cluster randomized trial. Overall, combined results from interviews and the feasibility implementation measure suggest the participants found the implementation strategy to be feasible. The results confirm the importance of carefully choosing the implementation team members and involving managers in the teams to enhance the implementation, as well as having a clear structure and support throughout the implementation phase. The implementation seminar was helpful to improve commitment among managers and provided them with important knowledge and helped to achieve a common understanding of implementation. Using already existing areas for training and competence enhancement of staff in the city districts were highlighted as a success, as this reduces unnecessary use of time and resources. As it is challenging keeping full attention on fall prevention over time among staff, repeating the focus regularly was described as necessary to succeed with the implementation.

The implementation strategy was useful and made sense of helping the implementation teams to plan and carry out an implementation process, in accordance with their local needs. Achieving coherence and making sense of the new practice is presented as an important factor in the implementation process in NPT [31] and not achieving this is a crucial challenge [32]. Making sense of fall prevention was also an important motivational factor for staff, influencing their compliance to the task. In NPT, making sense of new practice is crucial for participants acceptability because it enables them to grasp its relevance, purpose, and potential benefits [33]. Despite not knowing what they were participating in within the first workshop, the implementation team quickly achieved understanding and acceptance of the implementation strategy. Commitment from the members of the implementation teams plays an important part for achieving acceptance and approval of the new practice [31]. Who was chosen as members in the implementation teams was important for creating a committed group to lead the implementation process. Metz and Barley emphasise the importance of including key personal and key stakeholders in the teams, as they represent different perspectives on the implementation [34]. Beside choosing the right members of the implementation team, it is important to make sure the members have time to prioritize the work implementation requires. In NPT the construct of cognitive participation reflects the efforts made by those participating in the implementation through their engagement in the process, which directly influences the success of implementation efforts [33]. Throughout the study, some team members dropped out of the team due to sickness or other engagements. Being more flexible in replacing these members could have improved the engagement of the remaining team members.

Despite being carefully chosen as a team member by the managers, the members received little information on what role they were about to get. Getting relevant information on what the implementation strategy was and what role the implementation team would have, could have made the process easier. Clarifying the importance of this preparation and information to team members should be a topic in information provided to managers in advance. Lack of information is a well-known issue in services and reflects an important part of changing behaviour in practice: people need to understand what the managers are communicating and why. Lack of clear information could also be a result of mangers not quite understanding the implementation strategy in advance either. We therefore recommend more information and support to managers on how to inform team members before agreeing to be a part of a team responsible for implementation.

Our study also showed how important it was that managers had knowledge about implementation and were involved in the implementation teams. We should not assume all manager have implementation knowledge and know how to support and facilitate implementation. For managers as well there is little time to reflect on previous implementation efforts. Active involvement of managers in the implementation process has, in other studies, been found important for accomplishing organisational change and illustrating the importance of the work [35]. Having managers who provide support and motivation for a shared vision of change has also been a facilitator for implementation of evidence-based practice [36, 37].

Within NPT the third construct of collective action reflects how the implementation strategy was executed, examining the practical aspects of the implementation [33]. Throughout the implementation process, both teams agreed the implementation strategy was not time consuming, despite the three workshops in the planning phase. Implementation activities were considered manageable and less time-consuming when carried out through already existing arenas within the organisation. Similar challenges with available resources have also been highlighted in other feasibility studies using NPT in the analysis. A study testing the feasibility of training practice nurses to deliver a psychosocial intervention for people with depression and long-term care revealed that practice priorities and available resources were the most challenging aspects of succeeding with the implementation [38]. As the population of older adults is increasing in Norway and a policy of ageing in place has been established by the Government, the homecare services might face capacity issues in the future [39], and prioritising the most effective use of staff and time could be necessary. To succeed with implementation in that context will require time, dedication, structure and effective implementation strategies. Participants' positive attitudes toward the implementation strategy were also demonstrated within the Feasibility Implementation Measure, where most of the implementation team members found the strategy to be implementable, possible, and doable. However, the participants also reflected on the importance of the support they had received through the study, implying that this was a crucial aspect of the participants' perception of the implementation strategy's feasibility. This reflects the fourth construct of NPT, revealing how participants value and consider the use of the strategy in their routine practice [33].

This study did not test the implementation of all the national recommendations for preventing falls among community-dwelling older adults, and we did not assess if the city districts accomplished their local aims. The city districts also chose to target certain recommendations and established different aims for the feasibility study. A larger study should strive to implement all the recommendations, or plan for this, as they collectively constitutes evidence-based fall prevention services.

### Methodological strengths and limitations

The main strength of this study was the use of different information sources. This allowed us to evaluate the feasibility of the implementation strategy from different perspectives and levels in the organisation. However, the qualitative measure of feasibility FIM had low response rate (66%), since some of the member of the implementation teams did not reply to the questionnaire. To reduce social desirability bias regarding FIM, the survey was anonymous and conducted electronically. To avoid bias in the qualitative interviews, the implementation team members were interviewed by members of the research group who had not been actively involved in the study. Since the managers selected staff for the interviews, we did risk ending up with those who were most positive. Overall, staff was positive, bud did also provide us with some negative reflections. The predominance of positivity in the study results could be a result of these city districts appreciating their need for implementation competence and support.

All interviews were conducted immediately after the study ended, which did not provide us with information about long-term experiences but did reduce the risk of recall bias among the participants. We consider the findings from our study to be valid for evaluating the feasibility of the implementation strategy, as the NPT is a theory that has been proven robust for evaluating implementation [16]. The implementation strategy might, however, not be feasible for implementing in other settings than in homecare services, as they are organised differently. Additionally, the competence of the two authors who facilitated the process was an important contribution to the satisfaction of the implementation teams. Without the support of the authors this implementation strategy might not have been feasible.

### Implications of findings

Our findings illustrate how the implementation strategy, using the NPT framework, can be feasible and can thus be used to inform the strategy on a larger scale. This is important, both to inform the upcoming cluster randomised trial of FALLPREVENT and to understand how the implementation strategy can become a part of normality in homecare services.

## Conclusion

This study enhances our understanding of the feasibility of an implementation strategy involving support, dedicated implementation teams and improved implementation competence in Norwegian homecare services. Members of the implementation teams should be carefully chosen and they need support through the implementation process. Choosing small implementation activities to enhance fall prevention competence among employees in homecare services is not overly time consuming.

### Supplementary Information

Supplementary Material 1.

Supplementary Material 2.

Supplementary Material 3.

## Data Availability

The dataset supporting the conclusions of this study can be obtain by contacting the first author, Siv Linnerud.
