# Supplementary material for: Feasibility of an implementation strategy for preventing falls in homecare services
Source: Implement Sci Commun. 2024 Jul 19;5:79. doi: 10.1186/s43058-024-00615-7 (PMC11264773; doi:10.1186/s43058-024-00615-7)
Supplement: Supplementary file 1 — Supplementary Material 1. [file 43058_2024_615_MOESM1_ESM.docx]

**Feasibility of an implementation strategy for preventing falls in home care services – a mixed-method evaluation**

**Interview guide individual interviews**

Welcome from the interviewer:

The aim of this interview is to explore your experiences and opinions on the implementation study done in the city district where you work. You have already red the information paper and signed an informed consent for participation. Just as a repetition, it is voluntary to participate, and you can withdraw you consent at any time. The session will be recorded, so please remember not to share sensitive information about persons not present.

Do you have any questions before we begin?

The district you work in has participated in a project over the past ten weeks to test a plan for implementing fall prevention work. Over the last five weeks, your department has had an additional focus on fall prevention.

**Questions for the leaders**

1. Can you tell me a bit about your experience participating in this project?
2. During this period, there has been a team or group working together. Can you share your experiences with that?
3. Can you talk about what your role in the implementation work has been?
4. Now, we are about to start doing this on a larger scale in the big study. If you were to give us advice based on your experiences, what would it be?

**Questions for staff members**

1. Can you tell me about what has happened in your area regarding fall prevention in the last five weeks?
2. What are your thoughts on what has happened in your area in the last five weeks?
3. Can you tell me about how you see your role in fall prevention work?
4. Many other municipalities will soon begin similar initiatives in their home-based services departments as part of a larger study. If you were to give them advice based on your experiences, what would it be?

Finally, the interviewer will provide a brief summary of the key points and check with the participant to ensure understanding. To open the floor for additional information, the interview concludes by asking, "Is there anything I haven't asked about that you think is important, or that you would like to add?"
