# Supplementary material for: Feasibility of an implementation strategy for preventing falls in homecare services
Source: Implement Sci Commun. 2024 Jul 19;5:79. doi: 10.1186/s43058-024-00615-7 (PMC11264773; doi:10.1186/s43058-024-00615-7)
Supplement: Supplementary file 2 — Supplementary Material 2. [file 43058_2024_615_MOESM2_ESM.docx]

**Feasibility of an implementation strategy for preventing falls in home care services – a mixed-method evaluation**

**Topic guide focus group interviews**

Welcome from the moderator: The goal of this focus group interview is to hear about your experiences with the study you've participated in. You have already red the information paper and signed an informed consent for participation. Just as a repetition, it is voluntary to participate, and you can withdraw you consent at any time. The session will be recorded, so please remember not to share sensitive information about persons not present.

Do you have any questions before we begin?

Now, let's start. First, could you introduce yourselves with your first name, age, and professional background, so we can identify each voice on the recording?

1. Firstly, how has it been to be a part of this project?
2. You've been a team/group working together. How did you become a group, and how has the composition of the group been?
3. How did you work together as a team/group?
4. The group also included a leader. Can you tell us about that?
5. The first part of the project was a planning phase with three workshops.
6. During the planning of the implementation, you used a tool, the "Word" document that you filled out along the way. How did it work to use that tool?
7. In the final phase of the project, you tried out parts of what you planned in the planning phase. Can you tell us about your experience?
8. You also had access to a toolbox containing support materials such as PowerPoint presentations, quizzes, reflection questions. Can you tell us about that?
9. What is the most important thing you have learned/experienced from being a part of this?
10. As we are about to embark on a larger-scale study in Norway, if you could give us advice based on your experiences, what would it be?

Finally, the moderator provides a brief summary of the key points and checks with participants to ensure understanding. To open the floor for additional information, the interview concludes by asking, "Is there anything we haven't asked about that you think is important or that you would like to add?"
